# Parvalbumin Neurons in the Basal Forebrain Projecting to the Mammillary Nucleus Ameliorate Age-Related Cognitive Decline

**DOI:** 10.3390/ijms26135934

**Published:** 2025-06-20

**Authors:** Tingting Sun, Qianqian Li, Bimin Liu, Jiale Chen, Anan Li, Tao Jiang, Hui Gong, Xiangning Li

**Affiliations:** 1 MOE Key Laboratory for Biomedical Photonics, Wuhan National Laboratory for Optoelectronics, Huazhong University of Science and Technology, Wuhan 430074, China; suntingting@hust.edu.cn (T.S.); liqianqian@hust.edu.cn (Q.L.); chenjiale96@hust.edu.cn (J.C.); aali@hust.edu.cn (A.L.); huigong@hust.edu.cn (H.G.); 2 Key Laboratory of Biomedical Engineering of Hainan Province, School of Biomedical Engineering, Hainan University, Haikou 570228, China; liubimin8@hainanu.edu.cn; 3 HUST-Suzhou Institute for Brainsmatics, JITRI, Suzhou 215125, China; jiangtao@brainsmatics.org

**Keywords:** basal forebrain, parvalbumin neuron, vulnerable circuit, cognitive decline

## Abstract

Parvalbumin (PV) neurons in the basal forebrain (BF) orchestrate cognitive functions via extensive brain-wide projections. However, the age-related cognitive decline of their anatomical circuits remains poorly understood. Here, we employed viral tracing and fluorescence micro-optical sectioning tomography (fMOST) to reveal the vulnerability of the BF-PV circuits during aging. Quantitative whole-brain fluorescence intensity analysis revealed that BF-PV neurons projecting to the medial mammillary nucleus (MM) exhibited pronounced age-dependent neurodegeneration, characterized by 81.1% fiber loss and axonal swelling, while those innervating hippocampal CA1 showed a 70.3% reduction in fiber density. Optogenetic interventions demonstrated that selective activation of the BF^PV^-MM circuit can ameliorate cognitive deficits in old mice, significantly improving the novel object recognition index and its change rate. In contrast, modulation of the BF^PV^-CA1 circuit showed no significant effects. Moreover, with the whole-brain dataset, we reconstructed the morphology of individual neurons, revealing structural divergence between MM- and CA1-projecting PV neurons. Taken together, our results delineate the optogenetic-targeted activation of the BF^PV^-MM circuit, which can ameliorate age-related cognitive decline and provide both theoretical and therapeutic foundations for targeting neurodegenerative disorders.

## 1. Introduction

γ-Aminobutyric acid (GABA)-ergic neurons constitute the predominant population (50–60%) in the basal forebrain (BF) [1,2]. Studies have shown that BF-GABAergic neurons project to multiple brain regions [3], regulating fundamental physiological processes and critically supporting cognitive functions such as sleep–wake control [4], emotion [5], attention [6], and learning memory [7]. As the major GABAergic subtype in the BF [2], parvalbumin-positive neurons (PV) specifically express the calcium-binding protein parvalbumin, which tightly regulates intracellular calcium ion concentrations, thereby modulating neuronal excitability, synaptic plasticity, and neurotransmitter release dynamics—mechanisms essential for maintaining circuit functional integrity [8]. Specifically, BF-PV neurons utilize their fast-spiking properties to rapidly disinhibit target-projecting neurons and enhance oscillatory activities [9,10,11,12], which is conducive to the execution of cognitive functions.

Reduction of GABA-inhibitory synaptic transmission is associated with age-related cognitive decline [13], while PV neurons exhibit heightened vulnerability during healthy aging [14,15]. Numerous studies have shown that PV neurons exhibit high metabolic demands [16,17] to maintain a substantial amount of ATP for theta or gamma oscillating activities [9,10,11,12]. Once entering old age, PV neurons exhibit metabolic deficits, and circuit-specific degeneration may compromise cognitive processing, such as learning and memory.

BF-PV neurons exhibit extensive projections that connect multiple downstream targets across the whole brain [3,18]. Studies have shown that optogenetic activation of the terminals of BF-PV neurons in the thalamic reticular nucleus enables the promotion of arousal from non-rapid eye movement [19]. Moreover, regulation of the terminals of BF-PV neurons in the lateral habenula mediates reward and aversion [5]. The BF-PV neuron circuit projecting to dorsal CA1 modulates hippocampal supra-theta oscillations [11], which is related to learning memory [7,20]. BF-PV neurons also have fibers distributed in the medial prefrontal cortex that are involved in regulating the frequency of cortical gamma oscillations [9,10,12]. Additionally, regulation of the terminals of BF-PV neurons in the ventral tegmental area can induce social withdrawal [5]. Therefore, it should be emphasized that the BF-PV neurons regulate different functions in sleep awakening, reward aversion, learning and memory, and emotion through distinct projection pathways. However, the age-related degeneration of their anatomically distinct circuits remains poorly understood.

Therefore, it is necessary to assess the aging degree of different circuits, determine the circuit with the most significant changes, and then carry out optogenetic regulation, aiming to rescue the cognitive dysfunction in aged mice.

In this study, utilizing a combination of viral tracing and fluorescence micro-optical sectioning tomography (fMOST), we collected a 3D continuous dataset of the projection patterns of BF-PV neurons. Based on the fiber density and the axonal swelling in the targeting areas, we discovered two aging-vulnerable circuits, BF^PV^-MM and BF^PV^-CA1. Then, we used optogenetic intervention to confirm the different functions of these circuits. Our results show that activation of the BF^PV^-MM circuit can ameliorate age-related cognitive decline, while activating the BF^PV^-CA1 circuit does not lead to significant change. Single-neuron morphological reconstruction further revealed structural divergence between MM- and CA1-projecting PV neurons. This study provides an anatomical basis for exploring the dysfunction of BF-PV neurons and also provides new ideas for the treatment of neurodegenerative diseases.

## 2. Results

### 2.1. Vulnerable Projections of BF-PV Neurons in the Aging Mice

**Figure 1:**
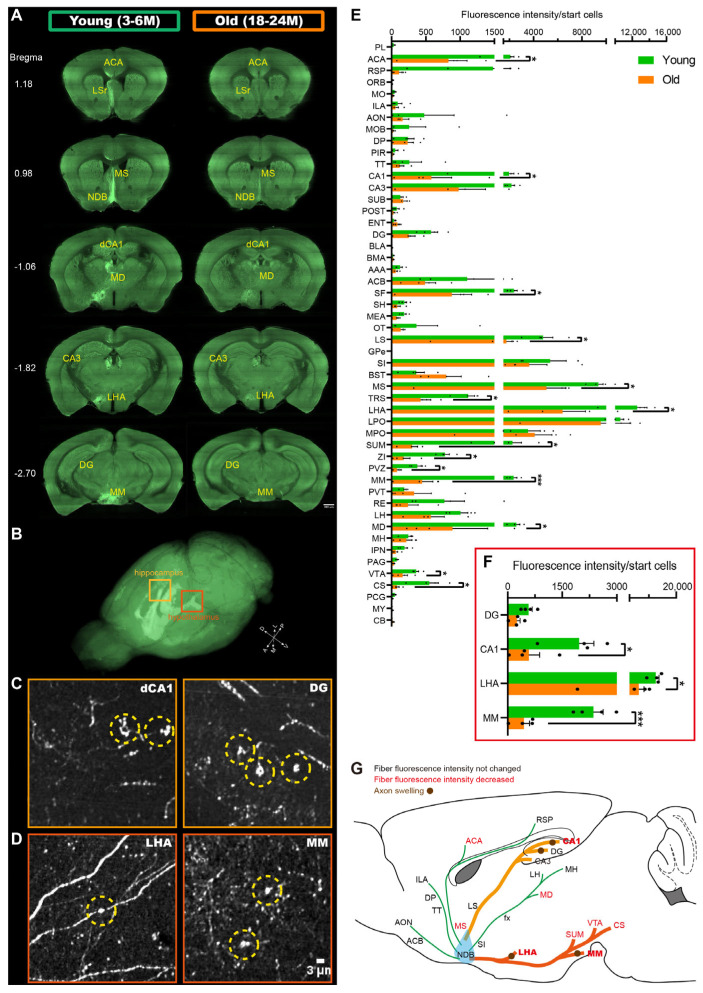
The aging degeneration of the whole-brain projections of BF-PV neurons. (**A**) The fluorescent image of BF-PV projections in the main targeting regions of the young group (3–6 months) and the old group (18–24 months), respectively. Scale bar: 200 μm. (**B**) With fMOST, the maximum intensity projection map of the fluorescence in the entire brain showed the axonal fiber distribution pattern of BF-PV neurons. (**C**) Axonal swelling was observed in the hippocampal subregions in the old group. Scale bar: 3 μm. (**D**) Axonal swelling in the hypothalamus. Scale bar: 3 μm. (**E**) The fiber fluorescence intensity of BF-PV neurons in multiple downstream target areas (from 4 mice in each group). (**F**) The changes in fiber fluorescence intensity in brain regions with axonal swelling. * *p* < 0.05, *** *p* < 0.001. (**G**) The degeneration degree of different BF-PV neuron circuits. Terms in black represent downstream target areas with no change in fiber fluorescence intensity. Pathways in green represent circuits that have not changed significantly. Terms in red represent downstream target areas with significantly reduced fiber fluorescence intensity. Brown circles represent axonal swelling. Pathways in yellow and orange represent circuits with severe degeneration.

BF-PV neurons have extensive projection across the whole brain, with fibers connecting to multiple downstream target areas. In order to identify the vulnerable circuits of BF-PV neurons, we used viral tracer with the fMOST to map the whole-brain projection pattern. In the coronal plane of several key projection targets, a reduction in the fibers of BF-PV neurons was observed in multiple downstream target areas, including the BF, hippocampus, and hypothalamus. We found a decrease in the fluorescence intensity of the fibers in these target areas, suggesting a reduction in the fiber density in the local regions (Figure 1A). In addition, the axon imaging results from fMOST (Figure 1B) revealed axonal swelling in the CA1 and dentate gyrus (DG) of the hippocampus (see yellow frame in Figure 1C), which was also observed in the lateral hypothalamic area (LHA) and MM of the hypothalamus (see orange frame in Figure 1D); no axonal swelling was found in other brain regions. We quantified the axonal swelling (diameters greater than 3 μm) in these four brain regions, as shown in the Supplementary Materials Table S1. This suggests that the degree of degeneration in different circuits of BF-PV neurons may have target preference. To quantitatively analyze the changes in the connection strength between BF-PV neurons and downstream brain regions, we statistically analyzed the fiber fluorescence intensities of 50 target regions across the whole brain. In order to eliminate the influence of the efficiency of viral infection, we first normalized the fiber fluorescence intensity with the initial cells. Our findings revealed a significant decrease in the fiber density of BF-PV neurons across the whole brain (Figure 1E). The fiber density decreased by 81.1% in the MM (*p* = 0.000825), 70.3% in the CA1 (*p* = 0.033621), and 49.1% in the LHA (*p* = 0.031473, Figure 1F). These regions correspond to the target regions where axonal swelling occurred. These results indicate that the BF^PV^-MM and BF^PV^-CA1 circuits have severe structural degeneration (Figure 1G).

### 2.2. Roles of BF^PV^-MM Projections in Cognition

**Figure 2:**
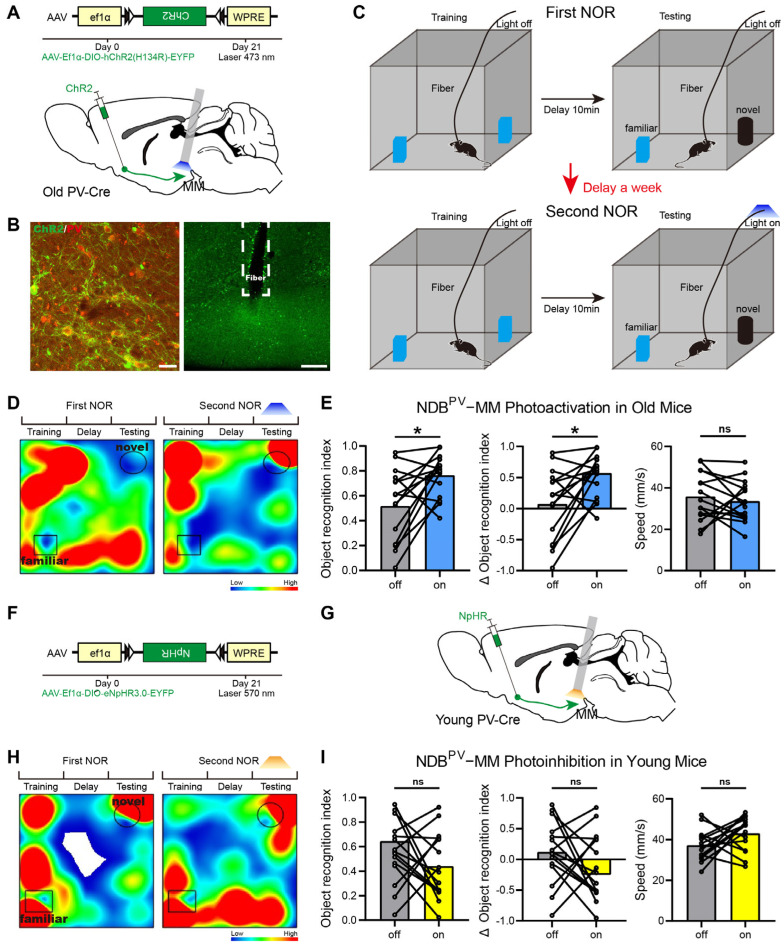
Roles of the MM in the cognitive function of BF-PV neurons. (**A**) Virus expression vector and schematic diagram of surgery used in the old group. (**B**) Virus injection sites and fiber embedding sites. Scale bar: 30 μm, 200 μm. (**C**) Operation procedures of optogenetic activation of the old group. (**D**) Time heatmaps of NOR in the old group. (**E**) In the old group, the effects of BF^PV^-MM activation on the recognition index of a novel object, index change rate, and movement speed (*n* = 15). (**F**) Virus expression vector and schematic diagram of surgery used in the young group. (**G**) Operation procedures of optogenetic inhibition of second NOR test in the young group. (**H**) Time heatmaps of NOR in the young group. (**I**) In the young group, the effects of BF^PV^-MM inhibition on the recognition index of novel object, index change rate, and movement speed (*n* = 15). * *p* < 0.05, ns represents no statistical difference.

To improve cognitive dysfunction in old mice, optogenetic regulation of BF^PV^-MM was implemented in accordance with circuit structure data. Our previous work found that optogenetic activation of the terminals of neurons can cause behavioral changes [21]. To investigate how the MM-projecting circuits participate in cognition, we performed optogenetic regulation to the PV terminals in the MM by injecting AAV-Ef1α-DIO-hChR2-EYFP in the BFs of old PV-cre mice (*n* = 15). The fiber was buried in the MM (Figure 2A). After 21 days of virus injection, we checked the expression of the virus and the location of the optical fiber embedding, and the slice proved that the strategy met the requirements of behavioral experiments (Figure 2B).

The behavioral experiment was conducted 21 days after virus expression. The novel object recognition (NOR) test behavioral paradigm was used to evaluate the cognitive levels of the mice. The mice were initially subjected to NOR without light stimulation to ascertain their baseline cognition level, and the behavioral data were meticulously recorded for each mouse. Subsequently, the mice were returned to their home cages to rest for a week, after which they were re-tested using a second NOR test with light stimulation to evaluate the impact of the light manipulation (Figure 2C). Between the two NOR tests, different sets of objects were utilized to prevent memory generalization. In the second NOR test, light stimulation (wavelength of 473 nm) was administered during the testing phase. We found that stimulating the BF^PV^-MM circuit increased the exploration time of the novel object in old mice (Figure 2D). Then, we calculated the behavioral indicators of each mouse before and after light stimulation. We found that the motor abilities of 15 old mice remained unaltered when BF^PV^-MM was activated. However, the recognition index for the novel object and the index change rate exhibited significant improvement (*p =* 0.0134, Figure 2E). This outcome signifies that the cognitive impairment of old mice has been improved to a certain extent. Optogenetic interventions demonstrated that selective activation of the vulnerable BF^PV^-MM circuit ameliorated age-related cognitive decline.

At the same time, we conducted a control experiment. In the young group, the virus AAV2/9-EF1α-DIO-NpHR-EYFPAAV-Ef1α-DIO-EGFP was injected into the BFs of 15 young mice (Figure 2F). The other experimental conditions were consistent with those described above, and light stimulation was also given in the testing stage of the second NOR test. In the second NOR test, light stimulation (wavelength of 570 nm) was administered during the testing phase (Figure 2G). Although the time spent by some young mice exploring new objects decreased (Figure 2H), the overall cognitive level of the 15 young mice remained unchanged (*p* = 0.2230, Figure 2I).

### 2.3. Roles of BF^PV^-CA1 Projections in Cognition

**Figure 3:**
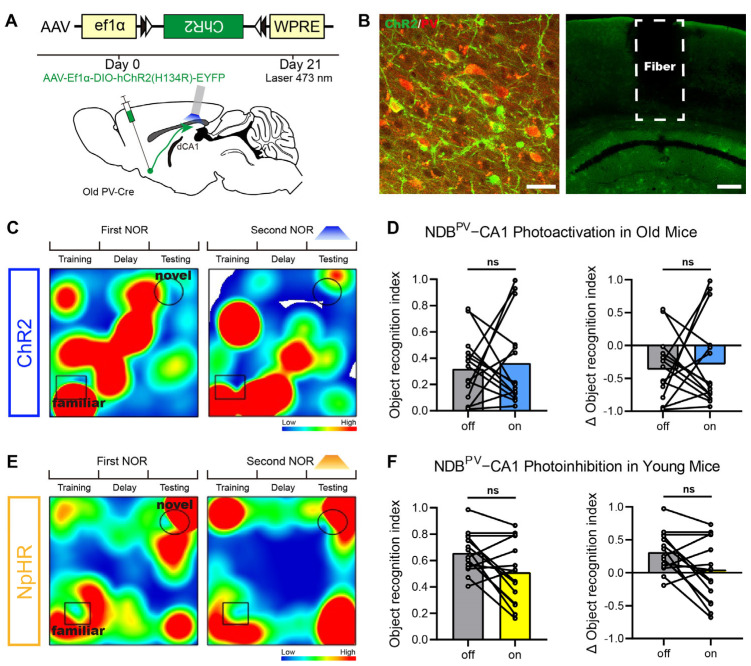
Roles of CA1 in the cognitive function of BF-PV neurons. (**A**) Virus expression vector and schematic diagram of surgery used in the old group. (**B**) Virus injection sites and fiber embedding sites. Scale bar: 30 μm, 200 μm. (**C**) Time heatmaps of NOR in the old group. (**D**) In the old group, the effects of BF^PV^-CA1 activation on the recognition index of novel object and index change rate (*n* = 15). (**E**) Time heatmaps of NOR in the young group. (**F**) In the young group, the effects of BF^PV^-CA1 inhibition on the recognition index of novel object and index change rate (*n* = 15). ns represents no statistical difference.

According to the anatomical results, the BF^PV^-CA1 circuit was degenerated, especially in the dorsal CA1 (Figure 1). To investigate how the CA1-projecting circuit participates in cognition, we performed optogenetic regulation to the PV terminals in the dCA1 by injecting AAV-Ef1α-DIO-hChR2-EYFP into the BFs of old PV-cre mice (Figure 3A). The fiber was buried in the CA1, and the other experimental conditions were consistent with those for MM-projecting neurons (Figure 3B). The experimental conditions were analogous to those described in Figure 2C, and light stimulation was administered during the testing stage of the second NOR test. When the BF^PV^-CA1 circuit was stimulated by light with a wavelength of 473 nm, the exploration of new objects increased in a few old mice (Figure 3C). However, the average exploration time of the 15 old mice did not change significantly. Furthermore, the activation of BF^PV^-CA1 in old mice did not elicit a significant change in the index change rate (*p =* 0.6777, Figure 3D). Optogenetic interventions, specifically the selective activation of the vulnerable BF^PV^-CA1 circuit, did not improve age-related cognitive deficits, although our study also inhibited the BF^PV^-CA1 circuit in young mice and some mice spent less time exploring the novel object (Figure 3E). However, the average cognitive level of the 15 mice showed no significant change after optogenetic inhibition (*p* = 0.0527, Figure 3F).

### 2.4. Heterogeneity of MM- and CA1-Projecting PV Neurons

**Figure 4:**
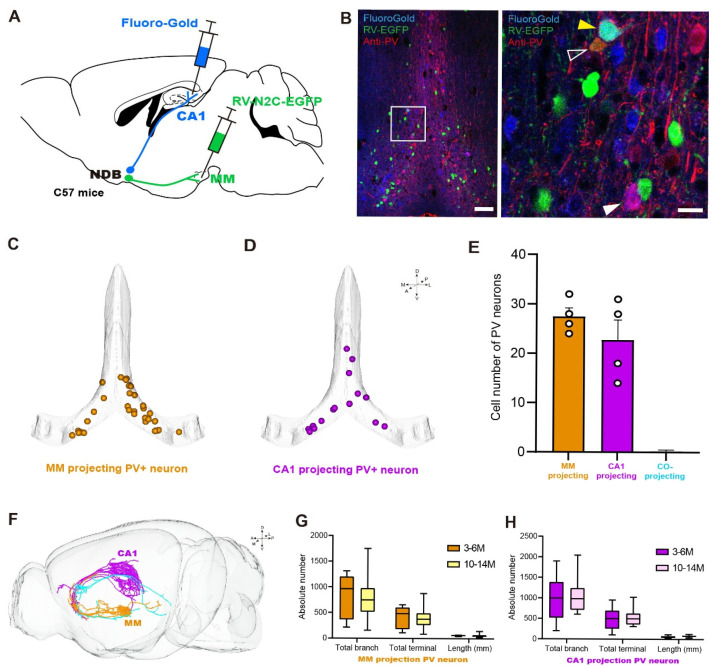
The morphology of BF-PV neurons projecting to MM and CA1. (**A**) The labeling diagram for MM- and CA1-projecting PV neurons with Fluoro-Gold and RV, respectively. (**B**) Distribution of neuron soma in the BF that project to MM (green), CA1 (blue), and PV positive (red). The white hollow arrow represents the MM-projecting PV neurons, and the orange one shows the overlap signals. The white arrow represents the CA1-projecting PV neurons, and the signals overlap and appear purple. The yellow arrow is the co-projecting PV neuron, and the signals overlap and appear cyan. Scale bar: 100 μm, 20 μm. (**C**,**D**) The soma location of MM- and CA1-projecting PV neurons. (**E**) The number of MM- and CA1-projecting PV neurons were counted (*n* = 4). (**F**) Single-neuron morphological reconstruction of MM- and CA1-projecting PV neurons, with the blue color denoting the co-projecting neuron. (**G**) Quantitative analysis of aging characteristics of MM- and CA1-projecting PV neurons: 3–6 M (*n* = 5); 10–14 M (*n* = 16). (**H**) Quantitative analysis of aging characteristics of PV neurons projected from the BF to MM: 3–6 M (*n* = 15); 10–14 M (*n* = 14).

To explore the circuit mechanism that causes functional differences, an analysis was conducted to determine whether the PV neurons that projected to the MM and CA1 were the same population of neurons. The upstream input of the MM and CA1 brain regions was analyzed using the combined labeling strategy of RV and FG in the same C57 mouse (Figure 4A). Then, the slices of the BF were recovered for the immunohistochemical staining of PV neurons (Figure 4B). The results showed that the PV neurons projected to the MM and CA1 were dispersed in the BF (Figure 4C,D), and quantitative statistical analysis revealed that only one PV neuron was found to be co-projecting (Figure 4E).

On the other hand, a Cre-dependent sparse labeling virus, CSSP-YFP-2E4, was utilized to obtain the single-neuron morphology of PV neurons. The morphological dataset for single neurons was obtained using the TDI-fMOST system. The proximal projection neurons were then filtered out. A total of 82 PV neurons were reconstructed. The PV neurons projected to the MM in the BF pass through the ventral side, while those projected to CA1 pass through the dorsal side. Notably, a single PV neuron was found to co-project (Figure 4F). These results indicate that the PV neurons projecting to these two target areas comprise two distinct populations.

This study aims to further investigate whether the morphology of these two groups of PV neurons also becomes heterogeneous during aging. However, the infection efficiency, limited by sparse labeling, was found to be extremely low in mice aged 18–24 months, precluding their use for morphological analysis. Therefore, a single study quantitatively analyzed the morphology of neurons in 10–14-month-old mice. The investigation revealed a decline in a number of morphological parameters (total branches, terminals, and lengths) of BF-PV neurons projecting to the MM or CA1 with advancing age. However, the decline was not statistically significant (Figure 4G,H).

## 3. Discussion

This study revealed the aging vulnerability of the BF neurons that play important roles in learning and memory. Optogenetic activation of the BF^PV^-MM neurons led to the amelioration of cognitive dysfunction in old mice, while the activation of BF^PV^-CA1 caused no significant improvement. With fMOST and retrograde labeling, we further discovered that PV neurons projecting to the MM and CA1 comprise anatomically and functionally distinct populations.

The BF-PV neurons directly project to different neurons in the hippocampus, including inhibitory interneurons, then regulate hippocampal theta oscillations and generate feedforward control between the BF and hippocampus [22]. However, the activation of the fibers of BF-PV neurons in CA1 did not result in a significant change in the object recognition ability of all old mice, suggesting the presence of alternative circuits that contribute to cognition.

Clinical studies correlate mammillary body atrophy (including the MM) with cognitive impairment, as patients with reduced volumes exhibit significantly lower memory scores, particularly in conditions like Alzheimer’s disease. This phenomenon mirrors laboratory studies. Moreover, the connection between the upstream brain region and MM is susceptible to environmental influences [23], resulting in the degeneration of the circuit structure. As one of the key components of cognitive circuits, the MM has long been thought to act as a repeater of hippocampus oscillation, transmitting information to the anterior thalamic nucleus and finally to the cingulate cortex, which are the core of the Papez circuit [24,25,26,27]. This view not only assumes that the MM has no independent role in cognitive function but also ignores the other input of the MM. In fact, the MM receives many non-hippocampal afferents [28,29], which can exert remote regulatory control over hippocampal and cortical activity by integrating and propagating input signals from the subcortex [30]. Consistent with previous studies, the present results demonstrate that BF-PV neurons directly project to the MM. Moreover, specific activation of BF-PV terminals in the MM instead of CA1 can ameliorate cognitive impairment in old mice. Our results further confirm that nerve fibers are prone to degenerative changes in the MM. Based on the projection preference of PV neurons, this study also revealed the high heterogeneity of BF-PV neurons. This was accomplished by using retrograde virus tracer and a sparse labeling strategy for single-neuron morphology. The results determined that the PV neurons projected to the MM and CA1 are two different populations. Single-neuron morphological reconstruction further unveiled structural divergence between MM- and CA1-projecting PV neurons.

This study has several limitations that should be addressed in the future. Firstly, the sparse labeling efficiency in mice aged 18–24M remains low, which may affect the accuracy and comprehensiveness of data acquisition. In the future, efforts can be made to address the issue of sparse labeling in old mice by designing more effective viral vectors. Secondly, the regulation of neural circuits was achieved through short-term interventions, potentially limiting our understanding of long-term functional changes. With the continuous advancement of biotechnology, the ability to conduct in-depth research on the regulation of long-term memory functions with specific labeling in different physiological states could open up promising avenues. These studies will help to a more comprehensive understanding of aging. Furthermore, the expressive ability of memory was detected during the testing stage of object recognition. Understanding the circuit’s role at different cognitive stages will deepen our understanding of the circuit and cognition. In the future, more behavioral paradigms can be utilized to explore behavioral changes in the training phase and delay phase of learning memory.

In conclusion, we discovered that the activation of the BF^PV^-MM circuit can ameliorate age-related cognitive impairment of object recognition. BF-PV neurons have functional heterogeneity. For example, adeno-associated virus vectors designed for gene therapy and targeting specific types of PV neurons may be conducive to the development of individualized medicine and have potential clinical value.

## 4. Materials and Methods

### 4.1. Animals

PV-IRES-Cre male mice and C57 male mice were obtained from Jackson Laboratory (Bar Harbor, ME, USA). Age classification was conducted according to Jackson Laboratory: young group (3–6 months), old group (18–24 months), and middle-aged group (10–14 months). All mice used in this study were housed in normal cages in an environment with a 12 h light/dark cycle with food and water ad libitum. All animal experiments were approved by the Animal Ethics Committee of the HUST-Suzhou Institute for Brainsmatics ([Hua]IACUC: S2021020).

### 4.2. Viral Vectors and Stereotaxic Injections

AAV2/9-EF1α-DIO-EGFP (4.2 × 10^12^ vg/mL), rAAV2/9-Ef1a-DIO-hChR2(H134R)-EYFP (2–2.5 × 10^12^ vg/mL), rAAV2/9-Ef1α-DIO-eNpHR3.0-EYFP (2–2.5 × 10^12^ vg/mL), RV-N2C-EGFP (3.00 × 10^8^ IFU/mL), and CSSP-YFP-2E4 (2–2.5 × 10^12^ vg/mL) were purchased from BrainVTA (BrainVTA Co., Ltd., Wuhan, China). Fluoro-Gold (FG) was purchased from Biotium (Biotium Co., Ltd., San Francisco, CA, USA).

The mice were intraperitoneally injected (100 g/mL) with sodium pentobarbital (1% wt/vol) before they were mounted and microinjected with a stereotaxic system. All virus injections were performed using a pulled glass micropipette at a speed of 15 nL/min and delivered with a micro-syringe pump (Nanoject II, Drummond Scientific, Broomall, PA, USA). Following the completion of viral injection, the needle was held for 10 min at the site and then retreated slowly. After that, incisions were stitched, and lincomycin hydrochloride and lidocaine hydrochloride gel was applied to prevent inflammation and alleviate pain for the mice.

For anterograde tracing, 50 nL of AAV2/9-Ef1α-DIO-EGFP was injected into the BFs (bregma 0.67 mm, lateral −0.6 mm, depth −5.4 mm from skull surface) of the mice (*n* = 4).

For the optogenetic experiment in the old group, 300 nL AAV2/9-EF1α-DIO-ChR2-EYFP was injected into the BF (*n* = 30). In order to manipulate the axon terminals of the BF-PV neurons, the optical fiber was planted into the MM (*n* = 15) or CA1 (*n* = 15) after the virus injection. The ceramic ferrule was supported with screws and dental cement fixed to the skull. The optical fibers (diameter, 200 μm, NA = 0.37, Newdoon Inc., Hangzhou, China) were implanted 300 μm above the CA1 (bregma −2.2 mm, lateral −1.1 mm, depth −1.73 mm from the skull surface) or MM (bregma −2.92 mm, lateral −0.2 mm, depth −5.4 mm from the skull surface). For the mice that were tested for fiber photometry or optogenetics, the dental cement and skull screws were applied to fix the optical fibers for further experiments.

For the optogenetic experiment of young group, 300 nL AAV2/9-EF1α-DIO-NpHR-EYFP were injected into the BF in the young group (*n* = 30). After the virus injection, the optical fiber was planted into the MM (*n* = 15) or CA1 (*n* = 15). All surgical procedures, experimental materials, and precautions are consistent with those used in the experiment involving old mice.

Additionally, in the control group (the virus without light-sensitive protein), 300 nL AAV2/9-EF1α-DIO-EYFP was injected into the BFs of old mice (*n* = 15). After the virus injection, the optical fiber was planted into the MM.

### 4.3. Anterograde Tracing and Array-fMOST Whole-Brain Imaging

For anterograde tracing, 50 nL of AAV2/9-Ef1α-DIO-EGFP was injected into the BF (*n* = 4). The mouse brains were obtained 21 days after virus expression. Then, the mice were intraperitoneally injected (100 g/mL) with sodium pentobarbital (1% wt/vol) and then perfused with 0.01 M PBS (Sigma-Aldrich Co., Ltd., St. Louis, MS, USA) for 10 min, followed by 4% PFA (Sigma-Aldrich Co., Ltd., St. Louis, MS, USA) for 10 min. Subsequently, the mice brains were removed and post-fixed in 4% PFA solution at 4 °C for 12 h. After that, the samples were embedded in agarose.

The imaging system mainly consists of a microscope and a microtome by Array-fMOST [31], with the optical microscope being composed of a line scanning confocal microscope. For whole-brain imaging, the virus-labeled and agarose-embedded samples were imaged with propidium iodide (PI) simultaneously staining cytoarchitecture landmarks. Fluorescence signals excited by two lasers at 488 nm and 561 nm are received by two sCMOS cameras through an array of optical elements for the rapid acquisition of green and red dual-channel data. The slicing device is composed of a high-precision vibrating slicing machine. In the process of data acquisition, when multiple scanning image strips can cover the entire sample surface, the program automatically collects the deep fluorescence signal layer by layer. Then, the vibrating microtome slices according to the section thickness set by the program. This image–slice–image cycle is repeated until all the data for the sample is obtained. Finally, a complete and continuous whole-brain dataset with a voxel resolution of 0.65 × 0.65 × 3 μm^3^ was obtained.

### 4.4. Data Processing and Registration

For the collected dataset, we first preprocessed the image to correct the uneven illumination and eliminate background noise [32]. The image stack of labeled outputs was registered to Allen CCFv3 using the transformation parameters [33]. We segmented several brain regions as landmarks through cytoarchitecture references, such as the outline, caudoputamen, medial habenula, lateral ventricle, and third ventricle. Based on these landmarks, we performed affine transformation and symmetric image normalization in Advanced Normalization Tools to acquire transformation parameters. Then, we extracted the fiber signals to obtain SWC files and continuous TIFF format pictures. After that, we imported the calculated datasets or images into Amira software (v5.2.2, Mercury Computer Systems, San Francisco, CA, USA).

### 4.5. Quantification

For the projection data, we employed the method from a previous study [34] to calculate the whole-brain projection of the BF-PV neurons. Briefly, a continuous whole-brain dataset was downsampled to a voxel resolution of 10 μm^3^ and then registered with CCFv3. Then, the datasets were segmented and binarized to extract the labeled signals. Next, we compared the extracted signals with the original data to remove the noise information. Finally, we obtained the signal points of data blocks at a volume of 1 μm × 1 μm × 1 μm and applied them to the whole-brain datasets to obtain the projection signals of different brain regions.

For two-dimensional slice cell recognition, the images were sequentially filtered with top-hat operation, binarization, and opening operations [35]. The resolution of the images used in this study was 0.65 μm/pixel; therefore, we chose a disk-shaped structure element with a radius of 9 pixels for the top-hat filter and 5 pixels for the opening filter. For binarization, the threshold depends on the signal-to-background ratio and the absolute intensity of the image, and we selected 30 as the fluorescence intensity threshold. When all slices were recognized and registered, the number of labeled neurons in each region was counted automatically. Then, we extracted the neuron signals to obtain SWC files, which were imported into Amira software (v5.2.2, Mercury Computer Systems, San Francisco, CA, USA) to achieve the visualization of the soma.

### 4.6. Behavioral Assays

The mice were used to conduct behavioral experiments between 8:00 AM and 6:00 PM. For all behavior tests, all the experimental mice were transferred to the behavior testing room a week prior to beginning of the first trial so that they could habituate to the conditions in the behavior testing room. The behavior chamber was cleaned with 75% ethanol prior to and after each test to remove any scent clues left by the previous subject mice.

### 4.7. Novel Object Recognition Test

All mice were individually habituated to the open field for 10 min for three consecutive days and then used in the novel object recognition (NOR) test. The NOR test includes three sessions of one trial each: training phase (memory acquisition), delay phase (memory consolidation), and testing phase (memory expression) trials. During the training trial, two identical objects are placed on opposite corners of the open field, mice were placed in an arena that contained two identical objects for 5 min, allowing them to freely explore the objects. Subsequently, the mice were returned to their home cages for memory consolidation (delay), and the testing session was performed 10 min after the acquisition trial. For the testing trial, one of the familiar objects presented in the first trial was replaced with a novel object. Then, the tested mice were placed back in the open field for 5 min, allowing them to freely explore two different objects. The mouse was scored for approaching the object when its nose was within 2 cm of the object, and the time spent in the exploration of each object was recorded. The total time spent in active exploration of the familiar (F) and novel (N) objects during the retrieval trial were calculated using VisuTrack (Xinruan Information Technology Co., Ltd., Shanghai, China) and analyzed using the novel object recognition index = N/(N + F) % and the delta recognition index = (N − F)/(N + F) %.

### 4.8. Optogenetics

The mice were first NOR tested without light stimulation to access their initial cognition level. Then, the mice were returned to their home cage to rest for a week and tested again using a second NOR test with light stimulation to evaluate the impact of the light manipulation.

The optogenetic manipulations were performed 21 days after the virus injection. The light stimulation was performed only in the testing phase of the second NOR experiment. The mice were coupled to a single-channel fiber optic patch cord connected to a 473 nm laser or 570 nm laser for optogenetic stimulation. The activation experiments in the old group were conducted with 15 ms pulse widths of 473 nm light at 20 Hz; the laser power was 10 mw. The photoinhibition experiments in the young group were conducted with 570 nm light (5 mw, persistent inhibition). The control experiment of optogenetics is detailed in Figure S1.

### 4.9. Retrograde Tracing

For retrograde tracing, 200 nL RV-N2C-EGFP was injected into the MM and 200 nL FG was injected at CA1 in the same C57 mouse (*n* = 4). The 7 days after injection, the samples were embedded in agarose.

### 4.10. Immunohistochemistry

For the immunohistochemistry experiment, the mice were perfused according to the steps described above and the samples were embedded in agarose. Then, the mouse brains were sectioned at a 70 μm thickness using a vibratome (VS1200S, Leica Camera AG, Wetzlar, Germany). The consecutive sections of BF were rinsed with 0.01 M PBS for 3 × 10 min and blocked with BSA (5% wt/vol) in 0.01 M PBS (at 37 °C for 2 h). Next, the brain sections were incubated with the following primary antibodies (at 4 °C for 12 h). The primary antibody in the retrograde tracing samples was anti-PV (MAB1572, 1:800, mouse, Merck KGaA, Darmstadt, Germany). Following the incubation with the primary antibody, the sections were rinsed and then incubated with the secondary antibody (1:800, at 37 °C for 2 h), which was Alexa Fluor-594 donkey anti-mouse antibody (A-21203, Thermo Fisher Scientific Inc., Waltham, MA, USA). Finally, the brain sections were attached to glass slides and imaged with a commercial confocal microscope (LSM710, Carl Zeiss, AG, Oberkochen, Germany).

### 4.11. Sparse Labeling and TDI-fMOST

In order to obtain the single-neuron morphology of BF-PV neurons, the sparse labeling virus dependent on Cre was selected, and 100 nL of CSSP-YFP-2E4 was injected into the BF. The mouse brains were obtained 21 days after virus expression, when they were embedded using HM20 resin.

The imaging system mainly consists of a microscope and a microtome and uses fluorescence micro-optical sectioning tomography based on a time-delay integration camera (TDI-fMOST) [32]. The embedded sample was fixed on a metal base, followed by the application of Na_2_CO_3_ solution. Under the action of alkaline buffer solutions, the surface fluorescent protein molecules undergo a chemical transformation and become fluorescent. If it is necessary to obtain the cytoarchitectural information of the sample, the appropriate PI solution is added. By moving the stage, the fMOST system imaged the surface of the sample in a line scan manner, then a diamond knife cut away the imaged surface. This allows the sample to be cut on the side. The process was repeated until the mouse brain was completely imaged. Finally, a complete and continuous whole-brain dataset with a voxel resolution of 0.32 × 0.32 × 1 μm^3^ was obtained.

### 4.12. Reconstruction and Visualization

For the morphological reconstruction of a single neuron in the whole brain, we employed the method from a previous study [36]. We first converted the collected high-resolution dataset into the Tdat format, imported it into Amira, applied the filament editor module to trace the neuron morphology, and saved the result in SWC format. To ensure the accuracy of the tracing path, each reconstructed neuron was manually verified. The reconstructed neurons were registered to CCFv3 based on the cytoarchitecture information, then the dendrites and axons of the registered neurons were split and imported into Amira software, respectively. The Identify Graphs interface was used to calculate the length, number of branches, and orders of axonal branches. NeuroGPS software (v.1.1.0) was used to identify and count the terminals in different brain regions.

The traced neurons were examined independently by three individuals to correct for possible errors in the reconstruction process. Afterwards, the registration method developed by our research group was employed and the reconstructed neurons were registered to CCFv3 using the cell architecture channel of the images. Subsequently, the dendrites and axons of the registered neurons were separated. The length, number of branches, and number of bifurcations of dendrites and axons were calculated using Amira software.

### 4.13. Quantitative Analysis

Statistical significance was analyzed using GraphPad Prism v.8.02. All measurements were listed as mean ± SEM. Statistical comparisons were performed using the multiple *t*-test or two-tailed Student’s *t*-test. Data were analyzed using Student’s *t*-test or the Mann–Whitney U-test when normality could not be assumed. Multiple *t*-tests were performed on data to assess the main effects of brain region and age. If significant differences were found, the False Discovery Rate was used to determine the source of the difference. Statistical significance was defined as *p* < 0.05, see Table S2 for details.

## Data Availability

The original contributions presented in this study are included within the article. Further inquiries can be directed to the corresponding author.

## Supplementary Materials

The following supporting information can be downloaded at: https://www.mdpi.com/article/10.3390/ijms26135934/s1.
