# Supplementary material for: Parvalbumin Neurons in the Basal Forebrain Projecting to the Mammillary Nucleus Ameliorate Age-Related Cognitive Decline
Source: Int J Mol Sci. 2025 Jun 20;26(13):5934. doi: 10.3390/ijms26135934 (PMC12250046; doi:10.3390/ijms26135934)
Supplement: Supplementary file 1 [file ijms-26-05934-s001.zip › ijms-3683857-supplementary.pdf]

Supplementary Materials

Table S1. The axonal swelling number of BF-PV neurons in old mice.

Region	Mice#1	Mice#2	Mice#3	Mice#4
DG	5	6	0	0
dCA1	7	6	6	2
LHA	0	0	2	0
MM	6	6	5	5

Table S2. The values of statistical analysis of results

Fig. 1F in MM	$t(6) = 6.181$	$p = 0.000825$
Fig. 1F in CA1	$t(6) = 2.743$	$p = 0.033621$
Fig. 1F in LHA	$t(6) = 2.793$	$p = 0.031473$
Fig. 2E	$t(28) = 2.640$	$p = 0.0134$
Fig. 2I	$t(28) = 1.246$	$p = 0.2230$
Fig. 3D	$t(28) = 0.420$	$p = 0.6777$
Fig. 3F	$t(28) = 2.023$	$p = 0.0527$

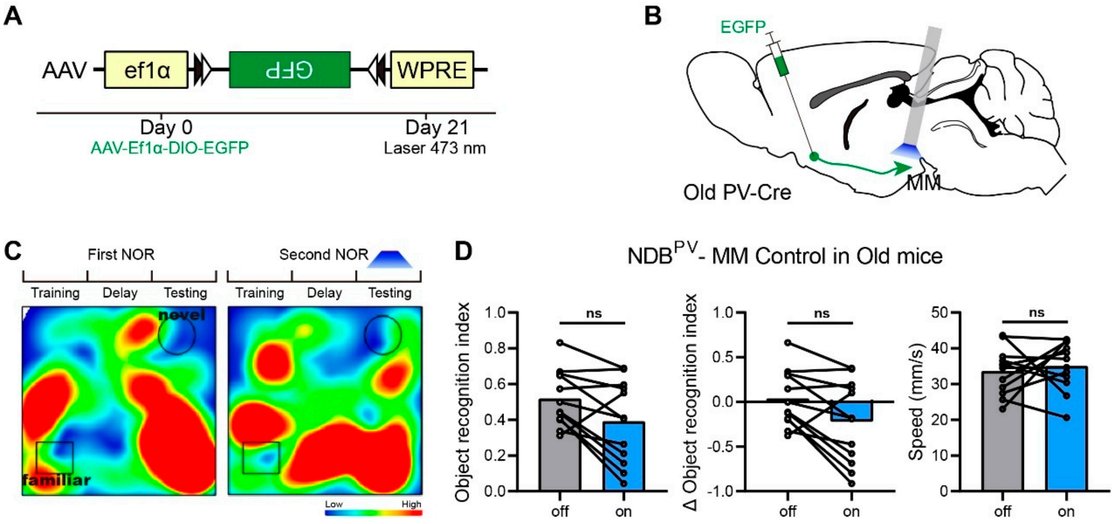

Supplementary Figure S1. Optogenetic activation of circuit BF^{PV}-MM in the old group without light-sensitive protein. (A) Virus expression vector and expression time used in the old group. (B) Schematic diagram of optogenetic activation in the old group. (C) Time heat maps of NOR in the old group. (D) The effects of activation BF^{PV}-MM on the recognition index of novel object, index change rate and movement speed in the old mice, n = 15.
